# Using Biocontrol Fungi to Control Helminthosis in Wild Animals: An Innovative Proposal for the Health and Conservation of Species

**DOI:** 10.3390/pathogens14080775

**Published:** 2025-08-05

**Authors:** Júlia dos Santos Fonseca, Beatriz Bacelar Barbosa, Adolfo Paz Silva, María Sol Arias Vázquez, Cristiana Filipa Cazapal Monteiro, Huarrisson Azevedo Santos, Jackson Victor de Araújo

**Affiliations:** 1 Department of Epidemiology and Public Health, Federal Rural University of Rio de Janeiro—UFRRJ, Seropédica 23890-000, RJ, Brazil; beatrizbacelar_@hotmail.com (B.B.B.); huarrisson@yahoo.com.br (H.A.S.); 2 Department of Animal Pathology, University of Santiago de Compostela—USC, Lugo, 27002 Galícia, Spain; adolfo.paz@usc.es (A.P.S.); mariasol.arias@usc.es (M.S.A.V.); cristiana.cazapal@usc.es (C.F.C.M.); 3 Department of Veterinary Medicine, Federal University of Viçosa (UFV), Viçosa 36570-900, MG, Brazil; jvictor@ufv.br

**Keywords:** *Duddingtonia flagrans*, nematophagous fungi, parasite control, wildlife, zoonoses

## Abstract

Helminth parasites of wild animals represent a major threat to the health of these animals, leading to significant losses in performance, health, and zoonotic implications. In some zoos, anthelmintics have traditionally been used to control these parasites, many of which are also zoonotic. Other actions, such as the removal of organic waste, have also been adopted. Few or no control measures are applied to free-ranging wild animals. Helminthophagous fungi are a promising biological alternative. When animals ingest fungal spores, they are excreted in their feces, where they trap and destroy helminth larvae and eggs, preventing and reducing the parasite load in the environment. Another alternative is to administer fungi by spraying them directly into the environment. This review aims to examine the use of helminthophagous fungi in the control of helminthiases in wild animals, highlighting their potential to minimize dependence on chemical treatments and promote sustainable animal breeding and production. There are many challenges to making this viable, such as environmental variability, stability of formulations, and acceptance of this new technology. These fungi have been shown to reduce parasite burdens in wild animals by up to 75% and can be administered through the animals’ feeding troughs. To date, evidence shows that helminthophagous fungi can reliably curb environmental parasite loads for extended periods, offering a sustainable alternative to repeated anthelmintic dosing. Their use has been linked to tangible gains in body condition, weight, and overall welfare in various captive and free-ranging wildlife species.

## 1. Introduction

Parasitic zoonoses are a critical component of global biosecurity at the wildlife–human–domestic animal interface. Accelerated urbanization, habitat fragmentation, and climate-driven extremes intensify helminth transmission among populations, necessitating integrated surveillance under the One Health paradigm [1]. In captive settings, such as zoos, rescue facilities, and urban green fragments, artificial host densities foster the accumulation of infective helminth stages in the soil, undermining classical sanitary strategies. A four-year longitudinal trial with captive ruminants showed that, despite successive deworming cycles, larval stages remained viable in the pasture, underscoring the limitations of conventional chemotherapeutic protocols [2].

Gastrointestinal helminthosis is of particular concern because its infective stages persist in the soil for extended periods and have a high zoonotic potential. Recent evidence from wild populations illustrates the extent of this challenge. For example, the detection of the neurotropic nematode *Baylisascaris procyonis* in Luxembourg, linked to the natural spread of invasive raccoons, confirms the species’ rapid European colonization and signals a substantial public health threat [3]. The risk becomes even more concerning when game species bridge rural and urban zones; for example, a 40% prevalence of *Ascaris suum* in Hungarian wild boars (*Sus scrofa*) demonstrates that these suids sustain peri-urban transmission cycles, contaminating soils also used by livestock [4]. The remarkable ecological plasticity of helminths is further highlighted by the wide diversity of *Ancylostoma* spp. detected in black bears, red foxes, and lynxes, showing that diverse trophic niches do little to limit their maintenance [5]. Long-term studies in European ruminants have revealed that *Trichuris* spp. remain viable even after several years of pasture rotation [6,7], underscoring the resilience of these parasites to conventional management strategies. Collectively, these findings support a common interpretation: wildlife populations harbor robust helminth reservoirs that challenge interventions based solely on chemotherapy, necessitating integrated strategies that consider the environment, multiple hosts, and public health. In developing countries, studies on zoonotic parasites in captive wild animals highlight the importance of implementing parasite control measures in environments in which humans, wild animals, and domestic animals interact. For example, a study conducted in Veracruz, Mexico, demonstrated this need in the context of captive wildlife [8]. Another study, conducted on parasites in wild rats in Saudi Arabia, located in Southwest Asia, indicates that wild animals, even in urban areas, can serve as important reservoirs of helminths of public health relevance, reinforcing the need for continuous monitoring and control [9].

**Figure 1:**
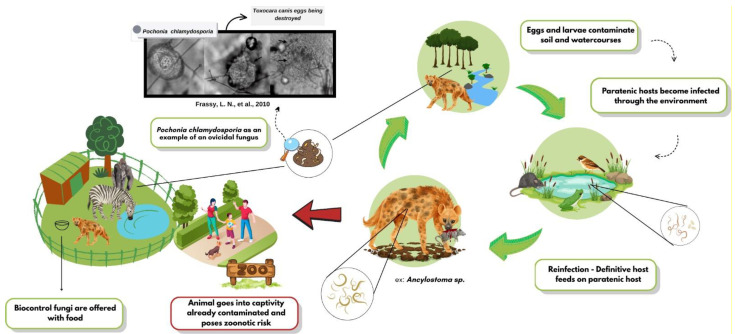
Dynamics of helminth biocontrol fungi in wild animals.

The growing resistance to chemical anthelmintics has accelerated the search for biological alternatives, and helminthophagous fungi have emerged as promising candidates. The high efficacy of genera such as *Duddingtonia*, *Arthrobotrys*, and *Pochonia* against various zoonotic helminths has been compiled, highlighting their sustainable potential [10]. The biocontrol dynamics of these fungi are shown in Figure 1, as well as the supply. In vitro assays have shown that *Arthrobotrys musiformis* combines physical trapping with lethal metabolites against *Haemonchus contortus* [11]. In field settings, spore formulations delivered in treats or edible gels reduced *Trichuris* spp. egg shedding by up to 90% in captive primates and bison [12]. Ten-month trials in wild ruminants have confirmed the effectiveness of combined ovicidal and larvicidal fungi [13]. These findings underscore the potential of multi-species approaches and novel delivery matrices for inclusion in management programs.

Against this backdrop, the present review compiles and critically analyzes the evidence on the use of helminthophagous fungi to control helminthosis in wildlife. Specifically, it aims to (i) synthesize laboratory and field data on the efficacy and mechanisms of action of these fungal species; (ii) examine ecological, technological, and regulatory factors that shape their performance across biomes and hosts; and (iii) identify knowledge gaps that can guide future translational research and inform management policies aligned with One Health principles.

## 2. Conservation of Wild Species

Research on the welfare of wild animals is essential for shaping biodiversity conservation strategies [14,15]. This effort encompasses both in situ studies conducted in natural habitats and investigations carried out in zoos and Wildlife Rehabilitation Centers, where high host densities promote pathogen circulation.

In zoological parks, the traditional exhibition role has increasingly given way to the preservation of threatened species and their health management [16]. Preventive measures include controlling gastrointestinal parasites with filamentous fungi isolated from the soil, which can diminish the infective stages in the environment [13].

Several studies have highlighted the practical value of these methods. *Mucor circinelloides* and *Verticillium* sp. had a fungal efficacy of 58% and 69% in inactivating *Toxascaris leonina* eggs eliminated in the feces of *Lynx lynx*. *M. circinelloides* and *Trichoderma atrobrunneum* reduced the viability of *Trichuris* sp. eggs in *Camelus dromedarius* feces by 50% [17].

Among wild ruminants in captivity, *M. circinelloides* and *Duddingtonia flagrans* effectively controlled nematodes with an efficacy percentage of 98% to 100% in the genera *Trichostrongylus*, *Nematodirus*, *Chabertia*, and *Haemonchus* for more than 3.5 years in species of the subfamilies Antilopinae, Caprinae, Bovinae, and Reduncinae [2]. The same pair of fungi was subsequently proven effective, between 69% and 71%, against trichostrongyles in *Cervus canadensis* [13].

Comparable results have been reported in studies on non-human primates. Gelatin-based treats containing chlamydospores of *M. circinelloides* or *D. flagrans* were offered to *Papio hamadryas*, achieving up to a 90% reduction in the shedding of *Toxocara canis* and *Trichuris* sp. eggs [18]. Finally, the same fungal combination achieved a comparable effect against *Trichuris* sp. in captive *Bison bison* [12].

Despite these advances, the ecological and behavioral heterogeneity of wild species limits the direct extrapolation of findings, underscoring the need for continuous research and systematic monitoring to support management plans that reconcile conservation, animal health, and biosecurity.

## 3. Helminths in Wild Animals

The presence of helminths in wild animals is natural and, at moderate burdens, usually remains in balance with their host. However, heavy infections cause lesions in the intestinal mucosa, impair nutrient absorption, and lead to loss of body condition in the host. In severe cases, they may result in infertility or death, thereby jeopardizing population viability [19]. Table 1 lists the principal helminth species reported in wildlife, their respective hosts, and those of particular public health relevance.

Growing anthropization is bringing humans closer to wildlife and their habitats, heightening the risk of parasite transmission among wild animals, domestic animals, and humans, and consequently increasing the prevalence of zoonotic diseases [38]. The association of zoonotic helminths with potential interrelationships among wild animals is more commonly observed in underdeveloped countries, in contrast to developed regions such as Europe, where parasite control is more effective and interactions between domestic and wild animals are significantly reduced [10,39].

## 4. Zoonotic Importance of Helminthosis in Wild Animals

Among all helminthiases affecting wild animals, soil-transmitted helminth infections, especially those caused by roundworms, are of particular concern. This large family of nematodes comprises more than 15 genera that affect wild animals and humans, as they are important zoonoses. Although each species has specific definitive hosts, species capable of becoming zoonotic agents and affecting humans have occasionally been identified [40]. Only the genus *Toxocara* was once considered zoonotic [41]; however, recent studies have shown that infections caused by *Ascaris suum* [22], *Toxascaris* [42,43], and *Baylisascaris procyonis* [24] can also occur. Humans can become infected by ingesting infective eggs or raw meat/organs from infected paratenic hosts or become accidental hosts by coming into contact with the infective stages, which migrate erratically within the organism as they are unable to complete their life cycles. After ingestion, the eggs hatch, and the larvae penetrate the intestinal wall and are transported via the circulation to various tissues (liver, heart, lungs, brain, muscles, and eyes). Human toxocariasis can be asymptomatic or present with different clinical syndromes depending on the affected organ, infection intensity, and host immune status. Allergic manifestations are common in patients with eczema [44]. Although the larvae do not undergo further development in these organs, they can cause local reactions and mechanical damage, leading to clinical toxocariasis [45,46,47]. Clear examples of these migratory patterns include visceral larva migrans syndrome (VLM), ocular larva migrans syndrome (OLM), and cutaneous larva migrans syndrome (CLM) [44,47,48,49], as well as neurotoxocariasis and covert (or common) toxocariasis [50]. Covert toxocariasis is clinically characterized in children by fever, anorexia, headache, abdominal pain, nausea, vomiting, lethargy, sleep and behavioral disorders, pharyngitis, pneumonia, cough, wheezing, limb pain, cervical lymphadenitis, and hepatomegaly. Common toxocariasis in adults is clinically characterized by weakness, pruritus, rash, breathing difficulty, and abdominal pain [51]. Toxocariasis can persist for several years as a chronic infection, and reactivation of encysted larvae can occur in immunocompromised individuals, leading to increased larval migration and exaggerated clinical symptoms [52]. To date, the most frequently detected and widespread zoonotic species is *Toxocara canis*. Zoonoses caused by *T. cati* remain controversial, and only a few infections have been reported, primarily in children with OLM. However, none of these infections have been confirmed using molecular techniques. Only one suspected case of *T. pteropodis* infection has been detected in humans, and this species has not yet been confirmed as a zoonotic pathogen.

Other genera of great importance in wild animals, as reported in several studies, include *Trichuris* spp. The incidence of *Trichuris* spp. in non-human primates is usually high, and *Trichuris trichiura* infects all primates, including humans [53]. Non-human primate species that can be infected include macaques, African green monkeys, baboons, squirrel monkeys, and woolly monkeys. Although mild infection is asymptomatic, severe infection produces clinical signs such as severe enteritis, anorexia, mucous gray diarrhea, and, sometimes, death [54]. Globally, *T. trichiura* remains one of the most important soil-transmitted helminths, along with *Ascaris lumbricoides* and hookworms [55]. To date, whipworms isolated from humans and other primates have traditionally been considered to be *T. trichiura* [56,57], whereas those recovered from pigs and wild boars are known as *T. suis* [27,28]. It is well known that differentiation between closely related *Trichuris* species is very difficult due to the phenotypic plasticity of the organisms themselves, host-induced variation, the paucity of morphological features, and the overlapping morphological characteristics that occur between species [58]. Thus, many studies on *Trichuris* have focused on the morphological and molecular differentiation of *T. trichiura* and *T. suis*, which are molecularly different but morphologically similar [28,59,60].

Nematodes of the family Ancylostomatidae (*Ancylostoma* spp. and *Uncinaria stenocephala*) cause cutaneous larva migrans in humans. *Ancylostoma* spp. eggs hatch in the soil, and infective larvae develop inside them. While oral ingestion of these larvae is the most common transmission route, they can also penetrate the human skin. Therefore, humans may experience itching due to larval movement, and secondary bacterial infections acquired by scratching are common. In massive infections, larvae can penetrate deeper tissues, leading to pulmonary and intestinal symptoms [29].

Among cestodes, *Dipylidium caninum* infections are common in carnivores [30,31,32]. Adult humans are rarely affected, as transmission occurs through inadvertent ingestion of fleas or lice infected with cysticercoids, and dipylidiosis is most often found in young children [61].

The family Taenidae, which includes the genera *Taenia* and *Echinococcus*, is responsible for other infections commonly found in carnivores [33]. Human infections with carnivore-specific metacestodes of different *Taenia* species are rare, although some cases of coenurosis caused by *Taenia multiceps* and *T. serialis* and cysticercosis caused by *T. crassiceps* and *T. martis* have been described [34]. *T. solium*, also common in pigs and wild boars, can lead to serious neurological conditions in humans and is responsible for brain damage that leads to epilepsy [62]. Cystic and alveolar echinococcosis, caused by *Echinococcus granulosus* and *E. multilocularis*, respectively, are considered among the most serious helminthic zoonoses because of their high pathogenic potential [33,61]. *E. multilocularis* has been described as an emerging threat to public health, as urban foci of infection have appeared in some European countries [33,63].

Zoonotic intestinal parasites are not the only concern; other worms can cause serious diseases in wild carnivores. Some of these parasites are a major concern for global health, as they are vector-borne zoonoses, such as heartworms *Dirofilaria immitis* [35] and *D. repens* [36], transmitted by culicids, and *Thelazia callipaeda,* whose vector is the fly of *Phortica variegata*.

There are several helminthic infections that can affect wild animals but are also of public health concern as they can affect humans, and their control is crucial from the One Health perspective. The presence of zoonotic helminths in wild fauna poses a risk to humans who share the same lands or are in close contact with them by visiting zoological parks.

## 5. Helminthophagous Fungi Useful for Controlling Helminths Affecting Wild Animal Species

The possibility of wild animals and livestock species sharing pastures increases the risk of helminth transmission [7,64]. Consequently, the usefulness of helminthophagous fungi in domestic animals can be extended to certain wild animal species. Table 2 summarizes the main endoparasites affecting both domestic and sylvatic animal groups.

All the above-mentioned helminths have in common that adult stages inside the hosts pass eggs by feces, and once in the soil, they evolve to the respective infective stages, mainly consisting of immobile phases, that is, eggs containing an inner larva, or mobile phases that exit the eggs and reach the third-stage larvae (L3) [88,89]. These are the targets for some species of saprophytic fungi that develop frequently in the soil and feed on organic matter, which also have the possibility of taking up carbon and nitrogen from some parasitic stages [90,91].

Most of the known species of helminthophagous fungi, or more generalist parasitophagous fungi, are filamentous species that develop mycelia that can act against eggs or larvae and exert different actions to take the inner content of the eggs and destroy the embryo. Consequently, this antagonistic activity relies on the prevention of infection through the reduction of the risk of animals accidentally ingesting them when grazing or feeding directly from the soil [17,44,57,92].

From a practical point of view, helminthophagous fungi candidates for controlling helminths of veterinary interest can be divided into two classes:

- Ovicide: As the name suggests, it groups fungi that are able to develop hyphae that can attach to the cuticle or the egg cover, penetrate inside, and take all the inner content. This is a large group, in which the most frequently cited species are *Pochonia chlamydosporia*, *Mucor circinelloides, Purpureocillium lilacinum*, and different strains belonging to the genus *Trichoderma* [93,94,95]. It is interesting to note that some of these fungi were tested against plant pathogens before their interest in veterinary medicine was discovered.
- Larvicide: In this case, the mobile phases or larvae are targeted, which is the reason for the numerous traps originating in their mycelia with the objective of trapping them; these fungi are called trapping or nematophagous fungi. The most representative species is *Duddingtonia flagrans*, which has been involved in more than half of the investigations performed up to now [96,97,98]. Another useful species belongs to *Arthrobotrys* spp., whereas it is suspected to belong to *Clonostachys rosea*.

Although another group of fungal antagonists of parasites can be found in the references, the endoparasitic fungi are of more interest, because it is required that “they are taken and brought inside the parasites,” which appears impossible in the case of the eggs and highly difficult for larvae. *Catenaria anguillulae* and *Harposporium anguillulae* are examples of these fungi [99].

The practical application of helminthophagous fungi for controlling helminths among sylvatic animal species is restricted to wild species kept in zoological gardens, principally confined to fenced plots where vegetation can grow during some seasons of the year [12,13]. The first reports refer to the administration of *D. flagrans* to giraffes maintained in a Florida Zoological Garden (Table 3) [100,101]. Some years later, different probes carried out in the Marcelle Natureza Zoological Park involved a great number of wild animal species infected by strongyles, roundworms, or whipworms, as summarized in Table 2. These investigations include the testing of *D. flagrans*, a mixture containing *M. circinelloides* and *D. flagrans*, and the less tested *Trichoderma atrobrunneum* or *Clonostachys rosea*. The good results obtained lead us to confirm the usefulness of these fungi in aiding the control of helminths affecting sylvatic animals, although the method of administration remains a pending question [2,13,17,40,102,103]. Regarding the distribution of these fungi, according to studies, they have been found in all regions of the world, from the tropics to Antarctica, in terrestrial and aquatic ecosystems, with a wide occurrence of these organisms in various natural environments, including extreme regions [104].

Finally, a very interesting advance was found in the possibility of simultaneously culturing different species of parasitophagous fungi, especially those with complementary activity, that is, ovicides and larvicides [105]. This discovery offers a useful strategy for controlling parasites that disseminate in different ways (eggs, larvae), and promising results have been demonstrated in horses, which leads to the consideration that it could be successfully applied to wild animal species [17].

## 6. Challenges for the Use of Helminthophagous Fungi to Control Helminthosis in Wild Animals

There are many challenges to making this viable, such as environmental variability, stability of formulations, and acceptance of this new technology, and therefore, preserving these species. The use of helminthophagous fungi reduces the need for anthelmintic drugs by offering a non-chemical alternative for controlling parasites. Research has shown that the secondary metabolites of the fungus *Pochonia chlamydosporia* exhibit nematicidal activity [106]. Virtually no control is applied to wild animals free in the wild.

The focus on “resistance,” “climate,” and “sustainability” suggests a growing interest in addressing long-term adaptation challenges and environmental viability. Fungi do not act immediately, like chemicals, and their action on helminth eggs and larvae in the environment can take time. There is no standard time for their effectiveness, and a higher density of animals with greater amounts of organic waste can prolong their action. Depending on the type of breeding, such as varying stocking rates, breeding environments, sanitization, and even the species of animals and their particularities, this can take a few months or even longer. Temperature extremes can affect the fungal growth, and some vehicles and abrasive substances can damage the fungus when exposed for a long time [107,108]. The fungus *Duddingtonia flagrans* grows best at temperatures between 25 °C and 33 °C [109]. There are only two commercial products for animal helminths worldwide, and they are not commercialized as they are on the European continent. It does not cover all continents, and with the legislation for biologicals in each country, it would not cover all zoos. These products are applied daily. This may cause resistance from producers, but in the breeding of these animals, where the risk of re-infection is high, the solution would be to mix it with feed and mineral salts. Although pelletized formulations containing helminthophagous fungi are not yet available in the veterinary market, they could enable less frequent dosing, as their slower gastrointestinal transit may eliminate the need for daily administration [107]. These pelletized formulations of *D. flagrans* have been successful, as seen in horses that were administered weekly doses for six months and had a reduction in their cyathostome parasite load of 82.5% and a weight gain of 38 kg compared with the control [110]. Some fungi are ovicidal, while others are larvicidal, and the association of fungi to increase their spectrum of action has been proposed and used in some studies [111]. Silver nanoparticles have also been proposed as promising tools and should be developed for future applications. A major challenge is to make nanoparticle-based formulations viable. The ovicidal activity of silver nanoparticles produced by the nematophagous fungus *D. flagrans* against *Toxocara canis* eggs was evaluated. There was destruction of up to 47% of the eggs and inhibition of development by 88% after 30 days [112]. Therefore, there are many variables to consider.

## 7. Conclusions

Field and laboratory evidence accumulated over the last decade shows that helminthophagous fungi can reduce environmental contamination by nematode eggs and larvae by 70–90% in a wide range of host species, including ruminants, equids, primates, carnivores, and bison, without the collateral effects associated with repeated anthelmintic chemotherapy. Their mode of action is multifaceted: species such as *Duddingtonia flagrans* and *Arthrobotrys musiformis* trap or penetrate infective larvae, whereas *Mucor circinelloides* and *Pochonia chlamydosporia* destroy eggs in the soil, offering complementary ovicidal–larvicidal coverage that can be combined in the same formulation for broader efficacy.

Beyond efficacy, fungal biocontrol aligns with conservation and One-Health principles by diminishing drug residues, curbing the spread of anthelmintic resistance, and lowering zoonotic risk at the wildlife–livestock–human interface. Long-term trials have demonstrated that regular administration through palatable carriers (e.g., gelatin treats or feed pellets) maintains low pasture infectivity and improves body condition scores and weight gain, thereby supporting both animal welfare and population viability.

Nevertheless, large-scale adoption still hinges on overcoming practical barriers, such as formulation stability under extreme temperatures and humidity, delivery logistics for free-ranging or semi-free animals, and heterogeneous regulatory frameworks governing biological products. Innovative approaches, such as pelletized or nanoparticle-based carriers that protect spores and co-culturing ovicidal and larvicidal strains to expand the spectrum of action, have shown promise in preliminary studies but require validation under diverse ecological conditions.

Therefore, we recommend that future research prioritize (i) the optimization of spore delivery systems suited to different husbandry regimes; (ii) multi-year, multi-species field trials that quantify ecological safety and cost effectiveness; (iii) harmonization of international regulations to facilitate product registration for zoological and wildlife settings; and (iv) greater control of access by humans and domestic animals to wildlife areas where wild animals live. Integrating helminthophagous fungi into broader parasite-management programs, together with habitat hygiene, rotational grazing, and targeted anthelmintic use, offers a realistic and sustainable pathway to safeguard wildlife health while protecting public health and environmental integrity. Furthermore, research aimed at producing fungal material in an economically viable manner is extremely necessary, in addition to being an important step towards enabling the commercial production of helminthophagous fungi.

## Figures and Tables

**Table 1:** Helminths identified in wildlife, their respective hosts, and their zoonotic significance.

Helminth	Wild Animals	Route of Infection	References
*Toxocara canis* * and *T. cati* *	Carnivores	Oral route, transplacental and transmammary transmission	[20,21]
*Ascaris suum* *	Pigs	Oral route	[22]
*Toxascaris leonina*	Carnivores	Oral route	[21]
*Baylisascaris procyonis* *	Carnivores (Raccoon) Primates	Oral route	[23,24]
*Trichuris vulpis* *	Canids	Oral route	[25]
*Trichuris ovis*, *Trichuris discolor*	Ruminants	Oral route	[6]
Trichuris trichiura *	Primates	Oral route	[26]
Trichuris suis *	Pigs and wild boar	Oral route	[27,28]
*Ancylostoma* spp. *	Carnivores	Oral route, percutaneous and transmammary transmission	[29]
*Uncinaria stenocephala* *	Carnivores	Oral route, percutaneous and transmammary transmission	[29]
*Dipylidium caninum* *	Carnivores		[30,31,32]
		Oral route—Ingestion of an infected flea	
*Taenia* spp. **	Carnivores	Oral route: Eggs—Intermediate hosts Contaminated meat—Definitive hosts	[33,34]
*Echinococcus granulosus* *, *E. multilocularis* *	Carnivores	Oral route: Eggs—Intermediate hosts Viscera of intermediate hosts—Definitive hosts	[33]
*Dirofilaria immitis* *	Carnivores	Vector-borne transmission	[35,36]
*D. repens* *	Carnivores	Vector-borne transmission	[36,37]

**Legend**: (*) indicates a zoonotic agent; (**) some non-zoonotic species.

**Table 2:** The main helminths that infect domestic animals and wild species.

		Helminths	Hosts		References
Group		Species	Domestic	Susceptible Wild Animals	
Trematodes					
		*Fasciola hepatica* Gastric fluke	Herbivores (Ruminants)	Herbivores (Ruminants)	[65,66,67,68]
Cestodes					
		*Taenia* spp. *Mesocestoides* spp. *Echinococcus* spp.	Carnivores (Dogs)	Carnivores (Wolves, foxes)	[69,70,71]
		*Moniezia* spp.	Ruminants	Ruminants	[72,73]
		*Anoplocephala* spp.	Equids	Equids	[74,75]
Nematodes					
	Trichostrongylids	*Trichostrongylus* spp. *Cooperia* spp. *Haemonchus* spp. *Teladorsagia* spp. *Marshalagia* spp.	Ruminants (Cattle, sheep, goats)	Ruminants (Moufflon, roe deer)	[76,77,78]
	Strongylids				
		Cyathostomins *Strongylus* spp.	Equids	Equids	[74,79,80]
	Hookworms				
		*Ancylostoma* spp. *Uncinaria* spp.	Carnivores (Dogs)	Carnivores (Wolves, foxes)	[70,81,82,83,84]
	Roundworms				
		*Toxocara canis* *Toxascaris leonina*	Carnivores (Dogs, cats)	Carnivores (Wolves, foxes)	[20,82,83,84]
		*Ascaris suum*	Swine	Swine	[4]
		*Toxocara vitulorum* (formerly *Neoascaris vitulorum*)	Ruminants	Ruminants	[73]
		*Baylisascaris procyonis*	Carnivores	Carnivores	[85,86,87]
	Whipworms				
		*Capillaria* spp. *Trichuris* spp.	All	All	[70,82,83]

**Table 3:** Control of helminths among wild animal species maintained in Marcelle Natureza Zoological Park (Outeiro de Rei, Lugo, Spain).

Helminth	Host	Parasitophagous Fungus	
Strongyles	Equines Lemur	*Duddingtonia flagrans* (larvicide)	66–94% 50–83%
Trichostrongylids	Wapiti, bison	*Duddingtonia flagrans* + *Mucor circinelloides* (ovicide) (blend)	69–71%
Trichurids	Bison	*Duddingtonia flagrans* + *Mucor circinelloides* (ovicide) (blend)	50%
Trichurids	Dromedaries	*Mucor circinelloides* *Trichoderma atrobrunneum* (ovicides)	50% 50%
*Toxascaris leonina*	Lynxes	*Mucor circinelloides* *Verticillium* sp. (ovicides)	58% 67%
*Baylisascaris procyonis*	Raccoon	*Duddingtonia flagrans* (larvicide) *Mucor circinelloides* (ovicide) *Pochonia chlamydosporia* (ovicide) *Purpureocillium lilacinum* (ovicide)	0% 53–69% 52–67% 45–62%

## Data Availability

Data sharing is not applicable to this article, as no datasets were generated or analyzed during the current study.
